# Reductive amination with zinc powder in aqueous media

**DOI:** 10.3762/bjoc.7.125

**Published:** 2011-08-10

**Authors:** Giovanni B Giovenzana, Daniela Imperio, Andrea Penoni, Giovanni Palmisano

**Affiliations:** 1 DiSCAFF, Università degli Studi del Piemonte Orientale “A. Avogadro”, Via Bovio 6, I-28100 Novara, Italy; Fax: +39-(0)321-375821, Tel: +39-(0)321-375846; 2 Dipartimento di Scienze Chimiche e Ambientali, Università dell’Insubria, Via Valleggio 11, I-22100 Como, Italy; Fax: +39-(0)31-2386449, Tel: +39-(0)31-2386234

**Keywords:** aldehydes, amines, reductive amination, water, zinc

## Abstract

Zinc powder in aqueous alkaline media was employed to perform reductive amination of aldehydes with primary amines. The corresponding secondary amines were obtained in good yields along with minor amounts of hydrodimerization byproducts. The protocol is a green alternative to the use of complex hydrides in chlorinated or highly flammable solvents.

## Introduction

Reductive amination is the name usually employed to indicate a synthetic protocol for the preparation of amines that involves a two-step reaction between a carbonyl compound and a primary or secondary amine (or even ammonia or ammonium salts), in the presence of a selective reducing agent [1–2]. The carbonyl compound and the nitrogen derivative undergo a dehydrative condensation to give an imine or an iminium ion, the latter being formally hydrogenated to the final amine product. The selectivity of the reducing agent is crucial as it should efficiently reduce the C=N bond of the intermediate while leaving unaffected the potentially reducible carbonyl compound. In addition, the desired reduction of the C=N bond may be accelerated by working in the presence of weak acids, thus allowing protonation of the C=N nitrogen atom but not of the carbonyl oxygen; the reducing agent, usually a hydride, should then be reasonably stable in the presence of acidic additives. Several reagents and combinations have been reported through the years; among them a predominant role has been played by modified borohydride reagents, such as NaBH_3_CN [3] and NaBH(OAc)_3_ [4–5]. Additional reducing agents may be exemplified by H_2_/cat [6–7], pyridine·BH_3_ [8] and picoline·BH_3_ [9], Zn(BH_4_)_2_/ZnCl_2_ [10], NaBH_4_/Mg(ClO_4_)_2_ [11], electrochemistry [12], 1,4-dihydropyridines [13–14], Ti(OiPr)_4_-PMHS [15], NaBH_4_-CoCl_2_ [16], iPrOH/RuCl(PPh_3_)_2_ [17], BH_3_·Me_2_S [18], PhSiH_3_-Bu_2_SnCl_2_ [19], and Zn/HCOONH_4_ [20].

Although the choice of reagents for reductive amination is quite large, a closer look from the viewpoint of green chemistry reveals that few of the above cited alternatives meet the requirements for sustainable processes [21–22]. Modified borohydride reagents are efficient and their activity spans a large array of substrates, but their atom economy is far from optimal; NaBH(OAc)_3_ offers a single hydride at the “price” of a molecular weight of 212, while NaBH_3_CN is plagued by the high toxicity of the cyanide that is potentially liberated during workup procedures. Moreover their use is nearly always limited to undesirable solvents: Carcinogenic 1,2-dichloroethane is the suggested first choice for NaBH(OAc)_3_ followed by the highly flammable tetrahydrofuran. Catalytic hydrogenation is an interesting alternative to modified borohydrides in terms of atom economy, but its application is unfortunately not general as many chemical groups must be avoided (e.g., double and triple bonds, nitro groups, benzyl–O and benzyl–N groups, etc.). Even electrochemistry, where the reducing agent (i.e., the electron) is the lightest, suffers from a limited case history and requires dedicated equipment.

Owing to the popularity of reductive amination in the preparation of amines, and its growing exploitation in industrial preparations, greener alternatives are required in order to reduce the environmental impact of the processes involving this useful reaction. Clearly, the use of water as a solvent is to be preferred and we were prompted to explore whether the elementary steps involved in reductive amination are compatible with aqueous conditions. A literature search revealed that imine formation is possible in water [23] and other green solvents [24]. The reduction of imine in aqueous conditions is reported for selected reagents. We were interested in the use of zinc metal in view of several factors promoting its use as a green reductant: i) atom economy, ii) low toxicity, iii) relative stability in air and water, and iv) low cost. Examples of the reduction of imines by zinc metal are reported, either in organic solvents [25–29] or in basic aqueous medium [30]. We recognized that this process could be considerably simplified by the development of a one-pot procedure involving the combination of a primary amine, aldehyde, and zinc in water, thereby avoiding the isolation of the intermediate imine. Although this combination appears to be plausible, its success is far from obvious: i) the reaction system is triphasic, resulting in complex and unpredictable phase dynamics of the involved chemical species; ii) the kinetics of the single processes may be mismatched, for example, imine formation may be slower than zinc oxidation by water; iii) carbonyl compounds are well-known to undergo different transformations under basic aqueous conditions, which may lead to extensive formation of byproducts.

## Results and Discussion

**Scheme 1:**
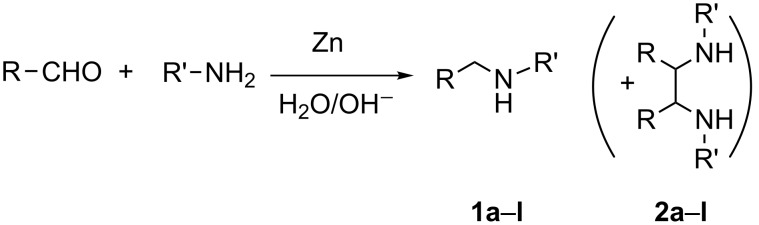
Reductive amination with zinc in aqueous base solution.

We investigated the reaction between benzaldehyde and benzylamine (Scheme 1, R = Ph R' = Bn) in 5% aqueous NaOH solution in the presence of zinc dust under vigorous stirring at room temperature. The reaction was complete within 24 h and standard work up afforded the desired secondary amine **1a** in 69–72% yield along with hydrodimers **2a** as byproducts (0–8%) (Scheme 1).

The use of an alkaline aqueous medium was necessary in order to fulfill the requirements of the reduction step, while the dehydrative condensation leading to aldimine is possible even in a neutral medium. Acidic pH is detrimental as the protonation of amine precludes imine formation; moreover, zinc dissolution is too fast under these conditions. The same reaction was then performed in the presence of alternative bases (LiOH, Na_2_CO_3_, NaHCO_3_, KOH, CaO), the best results being obtained with KOH (**1a**, 82%). Higher base concentrations (10% w/v) did not lead to significant improvements, while lower ones (1–2% w/v) strongly reduced the reaction yields. Finally, the effect of the reductant was studied: zinc dust (<10 μm, 98+% Sigma-Aldrich) was effective while other forms of zinc (foil, wire, mossy or shots) gave only minute yields, showing the surface-related nature of the reduction step, likely dependent on the extent of the imine adsorption. This was additionally supported by the fact that 15 equiv of Zn was the minimum excess needed to reach satisfactory yields, even though the recovery of unreacted zinc by filtration averaged about 80%. The recovered zinc powder was used for additional runs (after washing with water and drying in vacuo) and the consumed fraction replenished with “fresh” zinc powder. No difference in the reducing activity was noted in two reactions run with such recycled zinc powder. The scope of the reaction was assessed using different combinations of aldehydes and primary amines (Table 1).

**Table 1:** Results of the reductive amination with zinc in aqueous base solution.

	R^a^	R'^a^	Yield (**1**)^b^	Yield (**2**)^b^

a	Ph	PhCH_2_	82%	8%
b	2-Furyl	PhCH_2_	65%	7%
c	1-Naphthyl	PhCH_2_	48%	—
d	2-Naphthyl	PhCH_2_	55%	2%
e	Ph	*n*-C_4_H_9_	58%	7%
f	Ph	Allyl	60%	6%
g	*n*-C_5_H_11_	PhCH_2_	43%	—
h	*n*-C_5_H_11_	*n*-C_4_H_9_	33%	—
i	3-OH-Ph	4-OH-Ph	70%	<2%
j	3-OH-Ph	3-OH-Ph	65%	<2%
k	4-OH-Ph	4-OH-Ph	68%	<2%
l	4-OH-Ph	3-OH-Ph	55%	<2%

^a^See Scheme 1; ^b^Yields of isolated compounds.

In the first entries, benzylamine was chosen and fixed as the amine component, then different aromatic and heterocyclic aldehydes were employed, giving good yields of secondary amines along with minor percentages of coupling byproducts (except with 1-naphthalenecarboxaldehyde, where coupling is likely plagued by steric hindrance). These diamines likely arise from a mechanism involving dimerization of an intermediate aminoalkyl radical that is generated by electron transfer from the metal to the imine; this process is well-known and under different experimental conditions may predominate [31] and be useful for the preparation of 1,2-diamines [32]. Similar results were obtained upon reacting benzaldehyde with aliphatic amines (entries e and f). The reaction with allylamine allows us to appreciate the chemoselectivity of this protocol, leaving the C=C double bond untouched. Entries g and h rely on the combination of aliphatic substrates; the yields are lower and coupling byproducts were not detected, probably due to minor stabilization of aminoalkyl–radical intermediates involved in the reduction step. Additional examples (entries i to l) were explored with different combinations of isomeric hydroxybenzaldehydes and amines showing the successful reactivity of anilines and the compatibility of phenolic hydroxy groups.

The list illustrates the significant applicability of this one-pot zinc-mediated reductive amination. Nevertheless the protocol showed some limitations with selected substrates: i) Secondary amines failed to give tertiary amines; ii) hindered primary amines (e.g.*, tert*-butylamine and 1-aminoadamantane) did not react; iii) aminoesters reacted giving low yields of the corresponding carboxylic acid derivative (ester hydrolysis occurring even with *tert*-butyl esters).

## Conclusion

In summary, zinc dust in 5% aqueous KOH solution was found to be an environmentally friendly alternative to the large array of reagent/solvent combinations used for reductive amination. The attractive features of this method are high selectivity and ease of operation using cheap and safe reductants. Although the reaction is not as general as that with modified hydride reagents, a careful screening of the many variables involved in the reaction conditions will allow us to extend this protocol to a wider selection of substrates.

## Experimental

### Typical experimental procedure

In a round-bottomed flask the aldehyde (5 mmol) and the amine (5 mmol) were suspended in 25 mL of an aq. solution of potassium hydroxide (5% w/v), and zinc dust (<10 μm, 98+% Sigma-Aldrich) (15 equiv) was added. The mixture was vigorously stirred at rt for 24 h. Ethyl acetate (20 mL) was added and the residual zinc powder was filtered and washed with ethyl acetate. The filtrate and wash solutions were pooled and the organic layer was separated. The aqueous layer was extracted with ethyl acetate (2 × 20 mL), the combined organic layers were washed with water (2 × 20 mL), dried over sodium sulfate and evaporated under vacuum. The crude secondary amine was purified by column chromatography using petroleum ether/ethyl acetate.

### Characterization data

#### Dibenzylamine (1a)

^1^H NMR (300 MHz, CDCl_3_) δ 7.40–7.30 (m, 10H), 3.86 (s, 4H), 1.90 (bs, 1H); ^13^C NMR (75.4 MHz, CDCl_3_) δ 140.5, 128.6, 128.4, 127.2, 53.3; ESIMS *m*/*z*: calcd for C_14_H_15_N, 197.1; found, 198.2 (M + H)^+^.

#### *N*-Benzyl(furan-2-ylmethyl)amine (1b)

^1^H NMR (300 MHz, CDCl_3_) δ 7.40–7.24 (m, 6H), 6.34 (dd, *J* = 3.1, 1.8 Hz, 1H), 6.21 (dd, *J* = 3.4, 0.6 Hz, 1H), 3.80 (bs, 4H), 2.13 (bs, 1H); ^13^C NMR (75.4 MHz, CDCl_3_) δ 153.8, 142.0, 139.9, 128.6, 128.4, 127.2, 110.3, 107.2, 52.9, 45.4; ESIMS *m*/*z*: calcd for C_12_H_13_NO, 187.1; found, 188.1 (M + H)^+^.

#### *N*-Benzyl(naphthalen-1-ylmethyl)amine (1c)

^1^H NMR (300 MHz, CDCl_3_) δ 8.19–8.16 (m, 1H), 7.96–7.92 (m, 1H), 7.85 (d, *J* = 8.0 Hz, 1H), 7.60–7.33 (m, 9H), 4.31 (s, 2H), 3.99 (s, 2H), 1.87 (bs, 1H); ^13^C NMR (75.4 MHz, CDCl_3_) δ 140.5, 136.1, 134.1, 132.1, 128.9, 128.6, 128.5, 128.0, 127.2, 126.3, 126.2, 125.8, 125.6, 124.0, 53.8, 51.0; ESIMS *m*/*z*: calcd for C_18_H_17_N, 247.1; found, 248.4 (M + H)^+^.

#### *N*-Benzyl(naphthalen-2-ylmethyl)amine (1d)

^1^H NMR (300 MHz, CDCl_3_) δ 7.91–7.88 (m, 4H), 7.57–7.49 (m, 3H), 7.44–7.38 (m, 4H), 7.36–7.30 (m, 1H), 4.01 (s, 2H), 3.89 (s, 2H), 2.16 (bs, 1H); ^13^C NMR (75.4 MHz, CDCl_3_) δ 140.4, 137.9, 133.6, 132.9, 128.7, 128.4, 128.3, 127.9, 127.8, 127.2, 126.8, 126.7, 126.2, 125.7, 53.4, 53.3; ESIMS *m*/*z*: calcd for C_18_H_17_N, 247.1; found, 248.4 (M + H)^+^.

#### *N*-Butylbenzylamine (1e)

^1^H NMR (300 MHz, CDCl_3_) δ 7.37–7.20 (m, 5H), 3.78 (s, 2H), 2.63 (t, *J* = 7.2 Hz, 2H), 1.98 (bs, 1H), 1.56–1.45 (m, 2H), 1.41–1.28 (m, 2H), 0.92 (t, *J* = 7.2 Hz, 3H); ^13^C NMR (75.4 MHz, CDCl_3_) δ 140.4, 128.5, 128.3, 127.0, 54.1, 49.2, 32.2, 20.6, 14.1; ESIMS *m*/*z*: calcd for C_11_H_17_N, 163.1; found, 164.1 (M + H)^+^.

#### *N*-Allylbenzylamine (1f)

^1^H NMR (300 MHz, CDCl_3_) δ 7.38–7.08 (m, 5H), 5.89 (ddt, *J* = 17.2, 10.4, 6.1 Hz, 1H), 5.21–5.10 (m, 2H), 3.74 (s, 2H), 3.21 (d, *J* = 6.1 Hz, 2H), 3.00 (bs, 1H); ^13^C NMR (75.4 MHz, CDCl_3_) δ 139.6, 136.2, 128.6, 128.1, 127.2, 116.8, 53.0, 51.5; ESIMS *m*/*z*: calcd for C_10_H_13_N, 147.1; found, 148.1 (M + H)^+^.

#### *N*-Hexylbenzylamine (1g)

^1^H NMR (300 MHz, CDCl_3_) δ 7.36–7.15 (m, 5H), 3.79 (s, 2H), 2.63 (t, *J* = 7.2 Hz, 2H), 1.85 (bs, 1H), 1.50 (quint, *J* = 6.8 Hz, 2H), 1.37–1.18 (m, 6H), 0.89 (t, *J* = 6.1 Hz, 3H); ^13^C NMR (75.4 MHz, CDCl_3_) δ 140.5, 128.5, 128.2, 127.0, 54.1, 49.6, 31.9, 30.1, 27.1, 22.7, 14.2; ESIMS *m*/*z*: calcd for C_13_H_21_N, 191.2; found, 192.2 (M + H)^+^.

#### *N*-Butylhexylamine (1h)

^1^H NMR (300 MHz, CDCl_3_) δ 2.48 (t, *J* = 7.5 Hz, 4H), 1.46 (b quint, 4H), 1.37–1.18 (m, 9H), 0.91 (t, *J* = 7.4 Hz, 3H), 0.88 (t, *J* = 7.6 Hz, 3H); ^13^C NMR (75.4 MHz, CDCl_3_) δ 51.6, 51.3, 30.4, 27.4, 25.7, 24.3, 21.6, 19.4, 13.1, 12.7; ESIMS *m*/*z*: calcd for C_10_H_23_N, 157.2; found, 158.1 (M + H)^+^.

#### *N*-(3-Hydroxybenzyl)-4-aminophenol (1i)

^1^H NMR (300 MHz, CD_3_OD) δ 7.15 (d, *J* = 8.3 Hz, 2H), 6.87 (t, *J* = 7.8 Hz, 1H), 6.72 (d, *J* = 6.6 Hz, 2H), 6.20–6.03 (m, 3H), 4.13 (s, 2H); ^13^C NMR (75.4 MHz, CD_3_OD) δ 159.0, 157.3, 151.6, 132.1, 130.6, 129.7, 116.1, 106.5, 105.2, 101.0, 48.5; ESIMS *m*/*z*: calcd for C_13_H_13_NO_2_, 215.1; found, 216.1 (M + H)^+^.

#### *N*-(3-Hydroxybenzyl)-3-aminophenol (1j)

^1^H NMR (300 MHz, CD_3_OD) δ 7.10 (t, *J* = 8.0 Hz, 1H), 6.89 (bt, *J* = 8.3 Hz, 1H), 6.83–6.79 (m, 2H), 6.67–6.61 (bd, 1H), 6.19–6.08 (m, 3H), 4.19 (s, 2H); ^13^C NMR (75.4 MHz, CD_3_OD) δ 157.8, 157.2, 149.7, 141.5, 129.5, 129.2, 118.3, 113.9, 113.5, 105.5, 104.4, 100.0, 47.6; ESIMS *m*/*z*: calcd for C_13_H_13_NO_2_, 215.1; found, 216.2 (M + H)^+^.

#### *N*-(4-Hydroxybenzyl)-4-aminophenol (1k)

^1^H NMR (300 MHz, CD_3_OD) δ 7.16 (d, *J* = 8.6 Hz, 2H), 6.72 (d, *J* = 8.6 Hz, 2H), 6.63–6.52 (m, 4H), 4.08 (s, 2H); ^13^C NMR (75.4 MHz, CD_3_OD) δ 156.9, 150.1, 142.6, 131.5, 129.6, 116.24, 116.21, 115.6, 49.8; ESIMS *m*/*z*: calcd for C_13_H_13_NO_2_, 215.1; found, 216.1 (M + H)^+^.

#### *N*-(4-Hydroxybenzyl)-3-aminophenol (1l)

^1^H NMR (300 MHz, CD_3_OD) δ 7.14 (d, *J* = 8.6 Hz, 2H), 6.90 (td, *J* = 7.6, 0.9 Hz, 1H), 6.74 (d, *J* = 8.6 Hz, 2H), 6.19–6.09 (m, 3H), 4.09 (s, 2H); ^13^C NMR (75.4 MHz, CD_3_OD) δ 157.7, 155.9, 150.3, 130.9, 129.6, 128.6, 114.9, 105.5, 104.1, 99.9, 47.2; ESIMS *m*/*z*: calcd for C_13_H_13_NO_2_, 215.1; found, 216.1 (M + H)^+^.
